# Letter to the editor regarding “efficacy and safety of platelet-rich plasma combined with hyaluronic acid versus platelet-rich plasma alone for knee osteoarthritis: a systematic review and meta-analysis”

**DOI:** 10.1186/s13018-022-03479-6

**Published:** 2023-02-01

**Authors:** Ruibang Wu, Qiujiang Li, Ganjun Feng, Limin Liu

**Affiliations:** Department of Orthopedic Surgery and Orthopedic Research Institute, West China Hospital, Sichuan University, Chengdu, Sichuan China

## Abstract

In this letter to the editor, we discuss the article by Zhang et al., published recently in the *Journal of Orthopaedic Surgery and Research*. The authors reviewed the efficacy and safety of platelet-rich plasma combined with hyaluronic acid versus platelet-rich plasma alone for knee osteoarthritis. Whether the authors' purpose in grouping was to investigate the role of hyaluronic acid in the treatment of knee osteoarthritis is a question we would like to raise. In terms of the methodology of the study, combining randomized controlled trials with cohort studies in this meta-analysis is a methodological error. Secondly, the study methodology of the four included randomized controlled trial studies lacked a clear method of randomization. In addition to this, the completeness of the search needs to be taken into consideration. Some of the results of this study were highly heterogeneous, and no sensitivity analysis or meta-regression was performed to further analyse the sources of heterogeneity. The above issues will affect the conclusions of the article, and we believe this needs further improvement and discussion.

Dear editor:

We read with great interest the meta-analysis by Zhang et al. [1]. Entitled “Efficacy and safety of platelet-rich plasma combined with hyaluronic acid versus platelet-rich plasma alone for knee osteoarthritis: a systematic review and meta-analysis.” We congratulate the authors for publishing their study in “*Journal of Orthopaedic Surgery and Research*”. However, after review this article, there are several issues that may impact the study conclusions. We seek certain clarifications from the authors.

Firstly, the authors systematically evaluated the curative efficacy and safety of platelet-rich plasma (PRP) combined with hyaluronic acid (HA) in the treatment of knee osteoarthritis (KOA), comparing with platelet-rich plasma alone. However, both groups of patients were given the PRP therapy. Can we understand that the authors' aim was to investigate the role of HA in the treatment of KOA? And the efficacy of HA in the treatment of KOA is clear, therefore I would like to request the authors what is the significance of such grouping?

Secondly, combining randomized controlled trials with cohort studies for statistical analysis in this meta-analysis is a methodological error [2]. Therefore, performing subgroup analyses may reduce the heterogeneity of the results based on study design.

Thirdly, although the authors included a total of nine RCT studies, four of them [3–6] are Chinese studies. And the lack of clarity in the randomization methods described in the articles and clinical registries of RCT trials, which make it difficult to ensure relatively high data quality.

Fourthly, the authors searched extensively for published literature through four electronic databases (PubMed, EMBASE, Cochrane Library and CNKI), but these databases did not appear to be sufficient to retrieve all eligible studies. Alternatively, other databases such as Medline, Wanfang, NLM gateway as well as unpublished data like grey literature, which may help to obtain a more comprehensive collection of eligible studies.

Fifthly, some results of the meta-analysis were highly heterogeneous, and sensitivity analysis or meta-regression were not performed to further analyse the sources of heterogeneity, which weaken the conclusions.

Therefore, we hope that the authors will correct the relevant issues pointed out, which will benefit the research community as a whole.

## Data Availability

Not applicable.
